# CBT-based psychological rehabilitation program for oncological patients

**DOI:** 10.1192/j.eurpsy.2022.376

**Published:** 2022-09-01

**Authors:** A. Juhász, Z. Horváth, T. Szekeres, G. Vizin

**Affiliations:** 1 Semmelweis University, 1st. Department Of Surgery And Interventional Gastroenterology, Budapest, Hungary; 2 Eötvös Lorand University Institute of Psychology, Department Of Personality And Health Psychology, Budapest, Hungary; 3 Semmelweis University, Department Of Clinical Psychology, Budapest, Hungary; 4 Eötvös Lorand University Institute of Psychology, Department Of Clinical Psychology And Addiction, Budapest, Hungary

**Keywords:** cancer, Adherence, CBT program, shame

## Abstract

**Introduction:**

Although the survival rate of cancer patients show an increasing trend due to more effective treatments plans, cancer mortality rates are still the highest in Hungary among EU countries. From a psychological perspective, undiagnosed psychological disorders, insufficient treatment, and also poor adherence to treatment are recognized factors behind the saddening mortality data.

**Objectives:**

This translational research study aims to measure adherence rates and the extent of different psychological factors (including well-being and shame), in order to shed light on the relationship of these factors, among the population of patients with breast cancer. The secondary objective of the study is to develop a cognitive behavioral therapy -based psychological rehabilitation program for oncological patients (CBT-OP).

**Methods:**

A total of 372 participants took part in our study, consisting of patients with breast cancer (n=70), clinical control subjects (n=200) and healthy controls (n=102). Data collection was conducted with convenience sampling and in an online questionnaire format. Data was analyzed with the IBM SPSS 22.0 software package, using analysis of variance (ANOVA), correlation analysis and moderation analysis.

**Results:**

There was a significant difference between physical health, mental well-being, stigmatization and symptoms of post-traumatic stress disorder in the three groups. The association between adherence and mental well-being was moderated by the extent of experienced shame.

**Conclusions:**

Our results draw attention to the effects of shame and well-being on adherence to cancer treatment plans. Based on these findings we developed CBT-OP program, based on evidence-based CBT methods, focusing on reducing the experience of shame and on strengthening self-compassion skills.

**Disclosure:**

No significant relationships.

